# Corrosion Inhibition Mechanism and Efficiency Differentiation of Dihydroxybenzene Isomers Towards Aluminum Alloy 5754 in Alkaline Media

**DOI:** 10.3390/ma12193067

**Published:** 2019-09-20

**Authors:** Jacek Ryl, Mateusz Brodowski, Marcin Kowalski, Wiktoria Lipinska, Pawel Niedzialkowski, Joanna Wysocka

**Affiliations:** 1 Faculty of Chemistry, Gdansk University of Technology, Narutowicza 11/12, 80-233 Gdansk, Poland; mateusz.brodowski96@gmail.com (M.B.); m96kowalski@gmail.com (M.K.); wiktorialipinska154504@gmail.com (W.L.); joanna.wer.wysocka@gmail.com (J.W.); 2 Faculty of Mechanical Engineering, Gdansk University of Technology, Narutowicza 11/12, 80-233 Gdansk, Poland; 3 Centre for Plasma and Laser Engineering, The Szewalski Institute of Fluid-Flow Machinery, Polish Academy of Sciences, Fiszera 14, 80-231 Gdansk, Poland; 4 Faculty of Chemistry, University of Gdansk, Wita Stwosza 63, 80-308 Gdansk, Poland; pawel.niedzialkowski@ug.edu.pl

**Keywords:** aluminum alloy, corrosion inhibitor, alkaline environment, impedance analysis, adsorption, dihydroxybenzene

## Abstract

The selection of efficient corrosion inhibitors requires detailed knowledge regarding the interaction mechanism, which depends on the type and amount of functional groups within the inhibitor molecule. The position of functional groups between different isomers is often overlooked, but is no less important, since factors like steric hinderance may significantly affect the adsorption mechanism. In this study, we have presented how different dihydroxybenzene isomers interact with aluminum alloy 5754 surface, reducing its corrosion rate in bicarbonate buffer (pH = 11). We show that the highest inhibition efficiency among tested compounds belongs to catechol at 10 mM concentration, although the differences were moderate. Utilization of novel impedance approach to adsorption isotherm determination made it possible to confirm that while resorcinol chemisorbs on aluminum surface, catechol and quinol follows the ligand exchange model of adsorption. Unlike catechol and quinol, the protection mechanism of resorcinol is bound to interaction with insoluble aluminum corrosion products layer and was only found efficient at concentration of 100 mM (98.7%). The aforementioned studies were confirmed with Scanning Electron Microscopy and X-ray Photoelectron Spectroscopy analyses. There is a significant increase in the corrosion resistance offered by catechol at 10 mM after 24 h exposure in electrolyte: from 63 to 98%, with only negligible changes in inhibitor efficiency observed for resorcinol at the same time. However, in the case of resorcinol a change in electrolyte color was observed. We have revealed that the differentiating factor is the keto-enol tautomerism. The Nuclear Magnetic Resonance (NMR) studies of resorcinol indicate the keto form in structure in presence of NaOH, while the chemical structure of catechol does not change significantly in alkaline environment.

________________________________________________________________________________________

## 1. Introduction

Aluminum is the most widely spread metallic element on Earth [1], owing to its unique physico-chemical properties, such as low weight, high thermal and electrical conductivity, high linear expansion coefficient, and good corrosion resistance. It is non-magnetic, non-toxic and may be subjected to repeated recycling [2]. Aluminum and its alloys have been used in almost all industries, in particular in mechanical engineering, the defense industry, aviation, shipbuilding, food and chemical industries, and many others. It is a strategic resource whose consumption is a measure of countries’ development and industrialization level. Over the past 50 years, the world production of aluminum has been constantly increasing, with the highest leap occurring in this millennium. Limited access to bauxite ores and their gradual depletion are the main obstacles in the further development of the aluminum industry. Constantly growing demand and utilization of aluminum requires more effective methods for its recycling or protection.

Aluminum and its alloys belong to the group of passivating metals, alongside titanium, chromium and high-alloy steels. Spontaneously formed native oxide layer on the aluminum surface is built primarily of aluminum oxide Al_2_O_3_. The layer is thermodynamically stable in the pH range between 4 and 9, where aluminum possesses the highest corrosion resistance. On the other hand, the passive layer is not thermodynamically stable in alkaline and acidic media. In the presence of hydroxyl ions, aluminum undergoes oxidation to form Al(OH)_4_^−^, according to the mechanism proposed by Macdonald [3]. One of the most commonly accepted aluminum corrosion mechanisms in alkaline media may be simplified to the form of equations (1–4) [4]. Other, more detailed mechanisms suggests involvement of intermediate products and/or passive layer [5,6,7,8,9,10].

Al → Al^3+^ + 3e^−^ (1)

Al^3+^ + 3OH^−^ → Al(OH)_3_ (2)

Al(OH)_3_ ↓ + OH^−^ → Al(OH)_4_^-^ (3)

The anodic dissolution is accompanied by cathodic process of water electrolysis with hydrogen generation, according to Equation (4):

2H_2_O + 2e^−^ → H_2_ ↑ + 2OH^−^ (4)

The problem of aluminum corrosion in alkaline media occurs in numerous practical cases, starting from its possible application as an anode material in energy storage devices, through pre-treatment processes prior to anodization or for aesthetic purposes, up to the alkaline character of various cleaning agents used on working elements and constructions [11,12,13,14]. One of the most frequently utilized methods for lowering the corrosion rate of aluminum in these environments is the application of corrosion inhibitors.

Various organic inhibitors have been reported to be efficient corrosion inhibitors of aluminum and its alloys. Their inhibition effect depends on the molecule structure, the functional groups being electron donors or acceptors, and the number of such groups per molecule [15,16]. It is worth pointing out that the most effective inhibitors are based on molecules containing heteroatoms, such as oxygen, nitrogen, phosphorus, sulfur and aromatic rings [17,18]. Carboxylic acids in particular have been shown as highly efficient corrosion inhibitors of aluminum and its alloys in aqueous alkaline environments [19,20,21,22]. Studies carried out on maleic, malic, succinic, tartaric, citric and tricarballylic acids have revealed changes in corrosion efficiency with the increased amount of –COOH groups and decreased amount of –OH groups within inhibitor molecule [20]. Similar reports involved restriction of aluminum corrosion in alkaline media by polyacrylic acids, where studies shown increase of inhibition efficiency with the increase of molecular weight of inhibitor molecule [23]. Compounds containing nitrogen and/or sulphur have also been proved to be efficient corrosion inhibitors, an example of which may be studies on 3-methyl-4-amino-5-mercapto-1,2,4-triazole (MAMT). Inhibitor molecules adsorption on protected metal surface occurs through amine and thiol functional groups [24].

Lashgari and Malek proved that phenol is a highly efficient corrosion inhibitor of aluminum [25]. Phenols are deprotonated in alkaline environments and transformed into inhibitory active forms of phenoxide and phenolate. Similar conclusions, based on theoretical studies, were later on drawn for p-phenol derivatives, where the author confirmed that the mechanism of inhibition relies on a complicated cycle of protonation/deprotonation of inhibitor molecules in the inner area of the electrical double layer [26]. The process mentioned above leads to local neutralization of corrosive factors and their electrostatic repulsion in the vicinity of an active metal surface. Corrosion inhibition efficiency of p-phenol derivatives depends on several factors, including electron density on oxygen and hydrogen atoms in hydroxyl group, charge transfer, the energy of interaction, molecular activity, electric dipole moment and Gibbs free energy of the dissolution process.

The attention of corrosion scientists is nowadays increasingly focused on application of corrosion inhibitors of natural origin, obtained in accordance with the principles of green chemistry. Green corrosion inhibitors in the form of plant extracts are eco-friendly, non-toxic and biodegradable in neutral environments [27,28,29,30,31,32,33,34,35]. For example, *Phyllanthus amarus* leaf extract offers nearly 75% efficiency in 2M NaOH solution [36]. The extract contains alkaloids, cyanogenic glycosides, flavonoids, carbohydrates, sugar, proteins, triterpenoids and steroids. Functional groups –OH, –NH_2_, -SH, present in mentioned above compounds and π-bonds are most likely responsible for inhibition efficiency of *Phyllanthus amarus.* On the other hand, *Piper longum* seed extract, with 94% efficiency at concentration of 0.4 g·L^−1^ in 1 M NaOH, contains piperine, piperlongumine, piplartine, piperlonguminine, piperundecalidinine and pipernonaline [37]. The high inhibition efficiency was explained with presence of N-heteroatoms and π-electrons in aromatic rings. The 92% inhibition efficiency of *Gossypium hirsutum* extract in 2 M NaOH most likely originates from presence of O, N or S in amino acids such as: cysteine, lysine, methionine, phenylalanine, arginine, threonine, tyrosine, tryptophan, valine, but also polyphenolic aldehyde and tannins [32]. The authors also observed that higher concentration of active substances in present in leaves rather than seeds of *Gossypium hirsutum.*

Green corrosion inhibitors in the form of extracts from natural products are characterized by a large number of chemical compounds. In such a complex mixture of potential inhibitory compounds it is particularly important to perform phytochemical studies in order to determine the active compounds and their mechanism of interaction, which in many cases appears to be an incredibly difficult task. Therefore, in order to avoid blind-picking during selection of natural extracts containing potentially efficient inhibitor compounds one must get to know in detail the mechanism of interaction of various types of functional groups with protected metal surface as well as how it is modified by other aspects of the molecule structure. A valuable approach towards more effective determination of the active inhibitory compounds may be found in designing the extraction process. Differentiation of the type of solvents or extraction conditions leads to selective extraction of certain groups of compounds. Ryl et al. [38] showed that preparation of bee pollen extracts through extraction in different solvents results in different corrosion inhibition efficiency towards AA5754 in bicarbonate buffer at pH = 11. It has been proved that these differences are caused by varying content of carboxylic acids and phospholipids, which acted as inhibitory active substances in bee pollen extracts. 

A certain group of phenol derivatives exhibits very high corrosion inhibition efficiency towards aluminum and its alloys. This group includes catechol, cresol, chlorophenol, resorcinol, nitrophenol and aminophenol [39,40]. Talati and Modi [39] suggested that –OCH_3_, –NH_2_ and –CH_2_CHCH_3_ functional groups lower the inhibition efficiency of phenol, while –OH, –Cl, –NO_2_ increase it. Furthermore, they suggested three different adsorption mechanisms, namely: electrostatic forces, the formation of chelating agents with aluminum ions or covalent bond formation. The authors also observed that the inhibition efficiency decreases with the increase of electrolyte alkalinity. The synergistic interaction of resorcinol with Zn^2+^ ions was further studied by Lakshmi et al. [40], which revealed significant increase in corrosion inhibition efficiency of aluminum. However, all the aforementioned studies were carried out using the gravimetric method, introducing significant uncertainty to the measurements. The formation of the insoluble corrosion products layer on aluminum surface hinders specific determination of weight loss of the analyzed samples.

Not only the type and the number of active functional groups but also their position in the molecule structure may have a significant influence on corrosion inhibition efficiency. The chemical structure of isomer molecules affects modification of their physic-chemical properties such as solubility, while steric hinderance may influence both kinetics and mechanism of the adsorption process. This subject has not been given sufficient attention in the corrosion research; however, several available reports prove the importance of substituents position in the molecule [41,42,43]. Fouda and Elasmy [41] presented their studies on phenylthiosemicarbazide derivatives as aluminum corrosion inhibitors in 2M NaOH, with efficiency ranging between 75.0% and 98.5%, depending on the derivative. Hassan et al. [42] confirmed that the efficiency of aromatic carboxylic acids depends on the number and position of carboxylic groups and the presence of other substituents in the aromatic ring. The increasing corrosion efficiency was as follows: benzamide < benzaldehyde < acetophenone < benzoic acid < benzophenone (99.99% efficiency). 

The electrochemical impedance studies on thiosemicarbazone interaction with aluminum alloys in 1 M HNO_3_ revealed an almost 20% higher inhibition efficiency offered by para-substituted compounds in comparison to ortho-substituted ones [44]. There is no general relationship, though. The search for corrosion inhibitors of mild steel in 1 M HCl revealed that ortho-nitroaniline and ortho-methyloaniline show higher corrosion inhibition efficiency in comparison with both meta- and para-substituents, but in the case of phenlylenediamine, meta-substituted functional groups offered the highest efficiency [45]. A similar observation was made on aminophenol-N-salicylidene isomers’ action towards zinc in 0.5 M H_2_SO_4_ [46]. The goal of this work is to evaluate the influence of position of hydroxyl functional groups within dihydroxybenzene molecule on the corrosion inhibition provided by the isomer towards aluminum in alkaline electrolytes. Dihydroxybenzene isomers (catechol, quinol and resorcinol) were utilized, as presence of hydroxyl functional groups is expected to provide reasonable inhibition efficiency in studied electrolytes. In our studies we have implemented newly developed instantaneous impedance tool, i.e., Dynamic Electrochemical Impedance Spectroscopy in galvanostatic mode (g-DEIS), which is capable of accurate and time-efficient determination of the adsorption mechanism differences [19,20,38].

## 2. Materials and Methods 

### 2.1. Materials

The studied material was aluminum alloy 5754, which had the following alloying additives (in wt.%): Mg 3.6, Fe 0.3, Si 0.3, Cr 0.1, Mn 0.5, Ti 0.1 and Cu 0.1. Cylindrical samples were cut from a rod and subjected to pretreatment procedure in the form of grinding and polishing, carried out on Digiprep 251 (Metkon, Bursa, Turkey) polishing machine. Samples were grinded on a waterproof abrasive papers SiC 600 and 1500, polished with diamond suspensions 6 and 1 µm and mirror-finished on 0.05 µm silica. Following polishing, samples were cleaned and degreased in acetone using ultrasonic cleaner (Polsonic, Warsaw, Poland). Samples were exposed to corrosion studies with 0.5 cm^2^ surface area.

**Figure 1:**
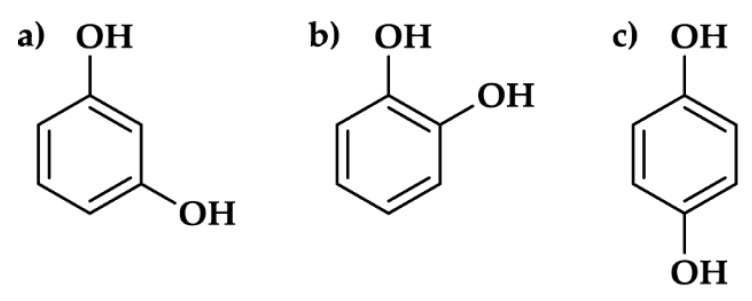
The chemical structure of dihydroxybenzene isomers: (**a**) resorcinol; (**b**) catechol; (**c**) quinol.

The primary electrolyte was the bicarbonate buffer solution. The buffer was prepared using 227 cm^3^ 0.1 M NaOH and 500 cm^3^ 0.05 M NaHCO_3_, diluted with deionized water to 1 dm^3^ volume. The obtained buffer had pH = 11 and conductivity of 3.8 mS·cm^−1^. All were analytical purity Sigma Aldrich reagents (Sigma Aldrich, St. Louis, MI, USA). Three dihydroxybenzene isomers were evaluated, namely: resorcinol, catechol and quinol. Their chemical structures are presented on Figure 1. The corrosion inhibition efficiency of the aforementioned compounds was investigated at various inhibitor concentrations c_inh_ = 1, 10 and 100 mM as well as with linearly changing inhibitor concentration during g-DEIS studies related to inhibitor injection into the corrosion cell.

### 2.2. Electrochemical Measurements

The electrochemical impedance studies were performed in a three-electrode setup. Investigated aluminum alloy 5754 was the working electrode (WE), Ag|Ag_2_O was the reference electrode (RE) (E° = +0.215 V vs. SHE) while Pt mesh served as the counter electrode (CE). The electrolyte volume in the corrosion cell was 10 mL.

Corrosion inhibition efficiency studies were performed by means of classic Electrochemical Impedance Spectroscopy (EIS) and Dynamic Electrochemical Impedance Spectroscopy in galvanostatic mode (g-DEIS) after the initial conditioning for 15 min. EIS was carried out in potentiostatic mode, at open circuit potential (OCP) conditions. The perturbation signal for EIS measurements was applied in the frequency range between 50 kHz and 40 mHz, with 10 points per decade of frequency and amplitude of 15 mV. Multisinusoidal perturbation signal for g-DEIS studies composed of 29 superimposed elementary signal in the frequency range between 4.5 kHz and 1.0 Hz, with 8 points per decade of frequency. Sampling frequency was 128 kHz. The amplitude of perturbation signal was controlled to assure the amplitude of response signal does not exceed 15 mV. The analysis window for the Short-time Fourier Transformation was 10 s in length. A similar measurement procedure was previously applied in corrosion studies [19,20,38,47,48,49].

**Figure 2:**
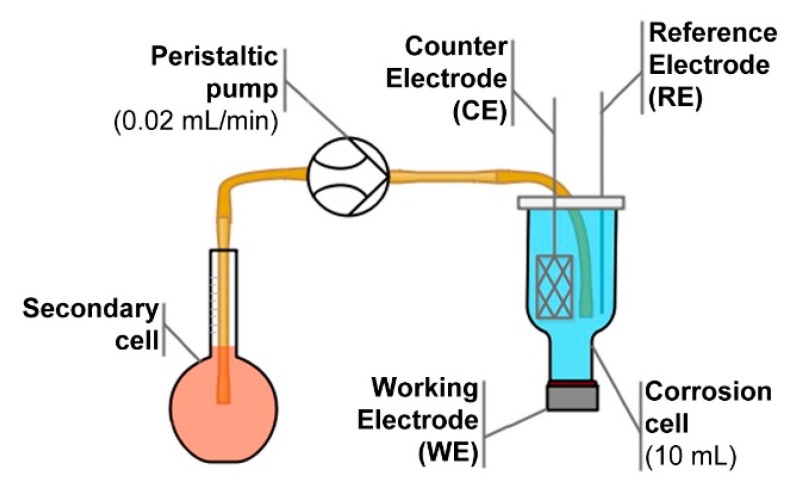
Schematic representation of the setup used during electrochemical studies.

To build the adsorption isotherm, studied corrosion inhibitor was injected from the secondary cell to the corrosion cell through BQ80S microflow peristaltic pump (Lead Fluid, Baoding, China). The flow rate was set as 0.02 mL·min^−1^. The concentration of studied inhibitor in the secondary cell was set in a way to assure corrosion inhibitor concentration in the corrosion cell (c_inh_) equal to 10 mM at the end of 6000 s long experiment. The corrosion cell was thermostated at 25 °C. The electrochemical setup used during all corrosion studies is schematically presented on Figure 2. 

### 2.3. Equipment

The EIS measurements were carried out using frequency-response-analysis on Gamry Reference 600+ potentiostat (Gamry Instruments, Warminster, PA, USA). The g-DEIS measurement system consisted of Autolab PGSTAT 302N (Metrohm, Herisau, Switzerland) galvanostat connected to PXI-4464 measurement card for AC signal generation and PXI-6124 card for AC/DC signal acquisition, both operating in PXIe-1073 chassis (all from National Instruments, Austin, TX, USA). The microflow peristaltic pump used was BQ80S (Lead Fluid, Baoding, China). The thermostat was Corio CD (Julabo GmbH, Seelbach, Germany)

Microscopy analyses of AA5754 corrosion process were performed on a Scanning Electron Microscope VP-SEM S-3400 N (Hitachi, Chiyoda, Japan), equipped with a tungsten source and operating at 20 kV accelerating voltage. SEM micrographs were done in secondary electron mode.

High-resolution X-Ray Photoelectron Spectroscopy (XPS) analyses were performed on Escalab 250 Xi multispectroscope (ThermoFisher Scientific, Waltham, MA, USA). The spectroscope is equipped in Al Kα X-ray source with a spot diameter of 250 µm. The measurements were carried out at 20 eV pass energy and 0.1 eV energy step size. The charge compensation was provided by means of low-energy electrons and low-energy Ar+ ions emission from the flood gun.

Nuclear Magnetic Resonance (NMR) spectra were recorded on AVANCE III 500 MHz NanoBay spectrometer (Bruker, Billerica, MA, USA). Tetramethylsilane (TMS) was used as an internal standard in all the measurements. The 0.5 mL solution containing 80 mM dihydroxybenzene isomer in D_2_O was filled with NaOH. The titrated compounds ratio to NaOH was 1:1, 1:5 and 1:10. ^1^H-NMR and ^13^C-NMR spectra were recorded. All the measurements were performed after 24 h in 25 °C and in the same volume of solvent.

## 3. Results and Discussion

### 3.1. Dihydroxybenzene Isomers as Corrosion Inhibitors

**Figure 3:**
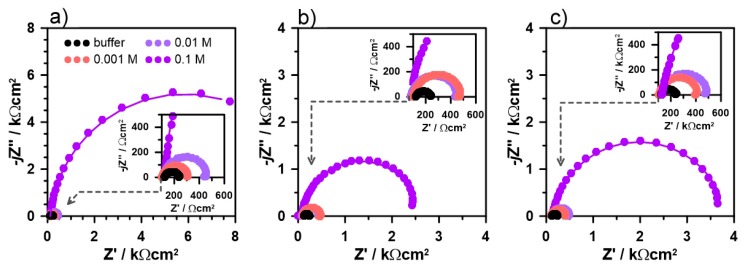
EIS impedance spectra recorded for AA5754 exposed to bicarbonate buffer (pH = 11) with the addition of the studied dihydroxybenzene isomers: (**a**) resorcinol; (**b**) catechol; (**c**) quinol at various concentrations in range 1–100 mM.

Electrochemical Impedance Spectroscopy (EIS) was used during the preliminary studies to assess the corrosion resistance of the investigated aluminum alloy immersed in an alkaline environment with the addition of each dihydroxybenzene isomer. The studies were carried out for three different corrosion inhibitor concentrations c_inh_, namely: 1, 10 and 100 mM. The impedance spectra, presented in the form of Nyquist plots, are plotted in Figure 3. It can be seen by the shape of the impedance semicircles, that studied derivatives offer different level of corrosion protection; however, detailed analysis required fitting of the obtained data with electric equivalent circuit (EEC). 

There is only one clear time constant present on the obtained impedance spectra, suggesting that the charge transfer through the electrode interface and through the adsorbed inhibitor layer are characterized with similar relaxation times. The authors decided to apply a simple form of Randles electric equivalent circuit (EEC) due to the prominence of one time constant on the impedance spectra in the applied frequency range. The one and only alteration was to replace the capacitance parameter with a constant phase element (CPE) in order to properly consider investigated electrode surface heterogeneity, originating from alloy microstructure and roughness, but even more important presence of local adsorption sites of corrosion inhibitor at its low concentrations. The CPE impedance is given with Equation (5).

(5) $$Z_{CPE}={\left[Q{\left(j\omega \right)}^{\alpha }\right]}^{-1}$$

It should be noted that in the boundary case, if α = 1, the CPE impedance responds to a capacitor of capacitance Q. Therefore, CPE exponent α is often considered as the homogeneity factor, its decrease represents the increase of surface heterogeneity, while Q reflects the quasi-capacitance of the heterogeneous electrode. Furthermore, the CPE can be used to estimate the effective capacitance of studied electrode C_eff_, with the use of Hirschorn’s approximation for the surface distribution of the capacitance dispersion [50]:

(6) $$C_{eff}=Q^{1/\alpha }{\left(\frac{R_{e}R_{ct}}{R_{e}+R_{ct}}\right)}^{\left(1-\alpha \right)/\alpha }$$

where R_e_ is the resistance of the electrolyte and R_ct_ is charge transfer resistance. The *χ^2^*-distribution of the selected EEC was in the range of 10^−4^, which is a good result, when taking into consideration EEC simplicity and system non-stationarity.

The shift in the calculated value of charge transfer resistance R_ct_ may be utilized to estimate the inhibition efficiency IE%, using the well-known relationship (7) [19]:

(7) $$IE_{\%}=\left(1-\frac{R_{ct}^{0}}{R_{ct}}\right)$$

where R_ct_^0^ denounces the measured value of charge transfer resistance in the absence of the inhibitor. The results of impedance data analysis with the R(QR) EEC are summarized in Table 1.

Interestingly enough, the addition of resorcinol at the lowest concentration (1 mM) provided the lowest level of corrosion resistance. This feature most likely originates from the altered mechanism of molecules adsorption on the surface of aluminum alloy and was an object of further studies. On the other hand, presence of catechol and quinol offers approx. 60% efficiency already at c_inh_ = 1 mM, which does not significantly improve until reaching substantially higher concentrations. This may be observed in particular through effective capacitance C_eff_ changes, which decreased by nearly 30% already at the lowest inhibitor concentrations, compared to reference buffer electrolyte. The most important factor affecting this parameter is the thickness of the adsorbed layer on aluminum surface [20]. 

Importantly, the highest inhibition efficiency was obtained after the addition of 100 mM of resorcinol. While the difference between IE% at this concentration does not exceed 4%, it should be noted that it corresponds to over 3× and nearly 5× higher R_ct_ values of AA5754 alloy in presence of resorcinol versus catechol and quinol, respectively. The increased efficiency of resorcinol at very high inhibitor concentrations was previously revealed in gravimetric studies [51]. In our opinion, its positive interaction is directly connected to the competitive formation of the corrosion products layer, making an additional barrier to aggressive environment. In the case of catechol and quinol, the barrier properties of the corrosion products layer are less evident, as discussed later.

### 3.2. The Adsorbed Layer Chemistry

The authors decided to focus on the core of electrochemical and physic-chemical studies on resorcinol and catechol isomers, which is due to the nearly identical response and adsorption mechanism between catechol and quinol. At the same time, there is a significant solubility difference between these two compounds in aqueous electrolytes at 25 °C, hindering possible utilization of quinol as efficient corrosion inhibitor. Quinol is sparingly soluble in water and show tendency for sedimentation over time. Its solubility in water is 5.9 g/100 g in comparison to 43 g/100 g for catechol and 110 g/100 g for resorcinol [52]. 

The chemistry of the adsorbed dihydroxybenzene layer and corrosion products layer on the surface of studied aluminum alloy 5754 was evaluated with the use of high-resolution XPS analysis. First, samples were pre-exposed to alkaline electrolyte with the addition of studied inhibitor at 10 or 100 mM concentration for a period of 24 h. The XPS spectra in the binding energy (BE) range of C1s, O1s, Al2p and Mg1s peaks were collected and deconvoluted using the fitting procedure described below. The results of the aforementioned deconvolution are summarized in Table 2.

**Figure 4:**
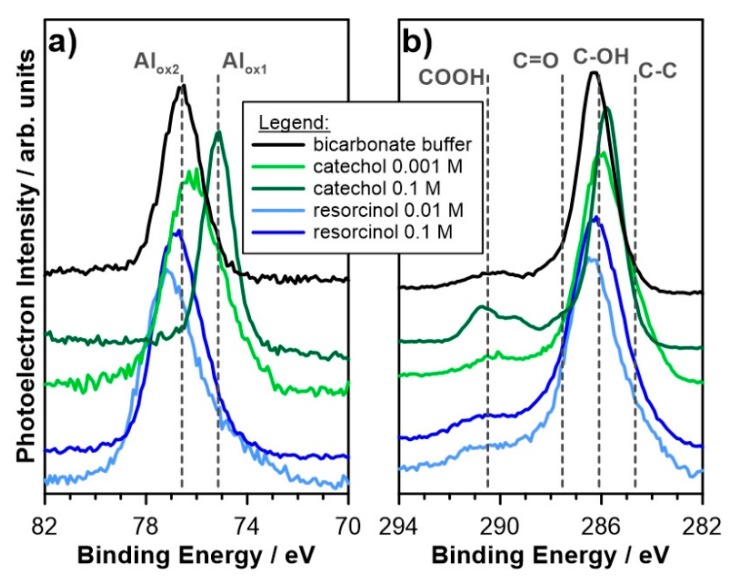
High-resolution XPS spectra recorded in (**a**) Al2p and (**b**) C1s binding energy range for aluminum alloy 5754 after 24 h exposure in bicarbonate buffer (pH = 11) and bicarbonate buffer with the addition of resorcinol or catechol at concentrations 10 and 100 mM.

Figure 4a presents the XPS spectra obtained in Al2p BE range. There is a clear shift in the peak position recorded between resorcinol-exposed and catechol-exposed aluminum samples. First, when exposed to bicarbonate buffer, but also with the addition of resorcinol, the primary peak doublet is located at higher BE values, with Al2p_3/2_ peak at approx. 76.7 eV. This component was labeled Al_ox2_ and ascribed to the non-stoichiometry aluminum corrosion product layer, often observed in studies of this metal in pH range 10–12 [53]. The second component (Al_ox1_), corresponding to native Al_2_O_3_ passive film was shifted at −1.9 eV. The presence of native oxide film results from air exposure of samples. The share of non-stoichiometric oxides (Al_ox2_:Al_ox1_) is nearly 13:1 when compared to native oxides for sample exposed in bicarbonate buffer, and drops to 5:1 after addition of resorcinol, regardless the concentration. The results suggest presence of corrosion products layer, which contributes the decrease of aluminum corrosion rate in these environments.

On the other hand, the addition of catechol at c_inh_ = 10 mM resulted in Al_ox2_:Al_ox1_ share of 1:3. The significantly lower contribution from Al_ox2_ may suggest that inhibitory action of catechol efficiently reduces formation of the corrosion products on aluminum alloy 5754 surface. This observation was confirmed for higher concentrations of catechol, where Al2p signal is composed solely of native oxide film. 

When compared to bicarbonate buffer exposed sample, the C1s spectra reveal the significant presence of carbon species for both analyzed dihydroxybenzenes and at both concentrations, suggesting that inhibitor molecules indeed take part in formation of the adsorbed layer on aluminum alloy 5754 surface (Figure 4b). The chemistry of this carbon species is strongly altered, however. In the case of catechol, the amount of C-C and C-OH species, based on peaks at 284.7 and 286.0 eV, respectively, is nearly 3× compared to resorcinol at the same concentrations [54,55,56]. Furthermore, the C1s spectra reveal significantly higher amount of carbonyl and/or carboxyl species for catechol-containing electrolyte (at BE exceeding 287.6 eV). These results corroborate previous findings regarding possible formation of the adsorbed inhibitor layer directly on aluminum surface and negligible participation of the corrosion products layer. On the other hand, in absence of corrosion inhibitor the C1s contribution originates primarily from C-OH and C=O species, testifying the interaction of bicarbonate species with aluminum sample.

The O1s spectra deconvolution is in good accordance with previously discussed results. In the absence of dihydroxybenzene isomers, over 47 at.% of the aluminum surface consists of oxygen atoms, with peak binding energies characteristic either for aluminum metal oxides (531.3 eV) or hydroxides (531.6 eV) [19,20]. Further on, this value slowly decreases for resorcinol-containing electrolyte, still within the range of 40 at.%. However, in the presence of catechol, the amount of O^2−^ species is significantly lower, at 10 and 100 mM. Here, the signal corresponding to peaks located at approx. 532.6 eV is still strong, but with an organic C-O interaction origin, instead. The last deconvoluted O1s component is located at binding energies exceeding 532.7 eV. Its source is adsorbed carboxyl species, but also chemisorbed water molecules [57]. The non-stoichiometric corrosion products layer reveals high hydration level, confirmed with SEM micrographs, therefore higher share of chemisorbed water on surface of bicarbonate buffer-exposed and resorcinol-exposed aluminum alloy 5754 is natural.

Finally, the amount of magnesium, the primary alloying additive, was only slightly altered between various investigated electrolytes. In each case magnesium was found in the form of hydroxides with Mg1s peak BE at 1303.1 eV, its higher contribution for resorcinol testifies the presence of magnesium oxides in the corrosion products layer, having a possible effect on the increased corrosion resistance [58].

**Figure 5:**
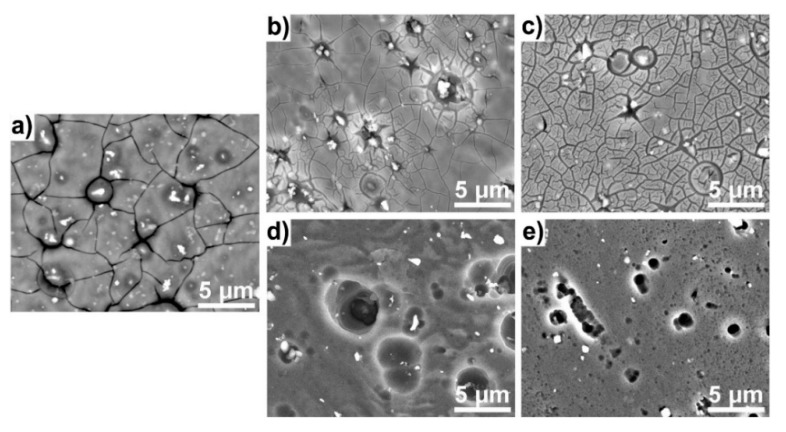
SEM micrographs of aluminum alloy 5754 surface exposed to bicarbonate buffer (pH = 11) for 24 h: (**a**) in absence of corrosion inhibitor; with addition of resorcinol at concentration (**b**) 10 mM and (**c**) 100 mM or with addition of catechol (**d**) 10 mM and (**e**) 100 mM.

The SEM micrographs shown in Figure 5 reveal significant difference in occurrence of the corrosion process for both studied dihydroxybenzene derivatives. The addition of resorcinol does not affect significantly surface topography when compared to reference aluminum alloy sample in buffer electrolyte (Figure 5a). The oxidation is primarily oriented around the alloy microstructure, which can be confirmed through local dissolution of alloy matrix surrounding intermetallic particles [58,59,60]. These particles appear on the micrographs with bright colors. Based on own studies and the literature survey discussed, the particles are primarily composed of aluminum and alloying additives of Fe, Cr and Mn—each cathodic in nature compared to metal matrix [60,61]. Furthermore, dense network of cracks visible on Figure 5a–c testifies high hydration level of adsorbed layer and corrosion products layer on the electrode surface. 

The presence of catechol or quinol in the electrolyte consequences in localized corrosion of aluminum, which is most often restricted to anodic Mg-rich phases and areas surrounding cathodic intermetallic particles. The absence of thick corrosion product layer effects in lack of cracks, which otherwise cover metal surface. The localized corrosion is naturally more evident at lower inhibitor concentrations (see Figure 5d), where the spherical shape of caverns should be connected with local areas of hydrogen evolution in coupled cathodic reaction [53].

### 3.3. Thermodynamics of the Adsorption Process

The thermodynamics of dihydroxybenzene isomers adsorption was evaluated based on instantaneous impedance studies, carried out in galvanostatic mode (g-DEIS). The studied inhibitor is injected with linear injection rate (0.02 mL·min^−1^); thus, the instantaneous value of inhibitor concentration in the corrosion cell is known. Dynamic impedance measurements allow for a precise evaluation of the instantaneous values of electric parameters, where the inhibition efficiency may be estimated from R_ct_ using previously introduced Equation (7). Studies carried out in galvanostatic mode at i_DC_ = 0 ensures constant measurement conditions and lack of an additional polarization component, resulting from corrosion potential changes during inhibitor injection. The approach is characterized with higher accuracy, allowing to obtain larger data set and evaluate the exact inhibitor concentration at which the linear character of the adsorption isotherm is modified due to full electrode surface coverage with inhibitor monolayer. The details of the experimental procedure are presented elsewhere [19,20].

**Figure 6:**
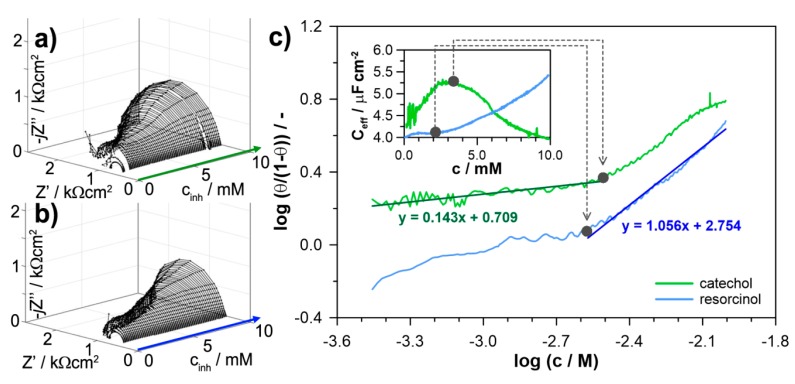
The g-DEIS impedance graphs of aluminum alloy 5754 in bicarbonate buffer (pH = 11), presented in Nyquist projection versus (**a**) catechol and (**b**) resorcinol concentration changes during its injection to corrosion cell. (**c**) Langmuir model of adsorption isotherms drawn based on instantaneous R_ct_ changes, in the inset the instantaneous changes of effective capacitance C_eff_.

The dynamic impedance spectra presented in the form of the Nyquist plot are shown on Figure 6a,b for resorcinol and catechol, respectively. The shape of the impedance spectra develops during corrosion inhibitor injection, where the increased impedance loop diameter testifies the increase of aluminum corrosion resistance. Fitting impedance spectra with R(QR) EEC allows for determination of dynamic changes of the electric parameters: R_ct_ and CPE within a timeframe of an analytical window length of a Short-Time Fourier Transform function, equal to 10 s in this case. The fitting procedure was carried out using dedicated software based on Nelder-Mead algorithm and build in LabView environment. The resultant χ^2^-distribution was typically in 10^−4^ range and did not exceed 2 × 10^−3^. Determination of the instantaneous R_ct_ values allowed for calculation of momentary inhibition efficiency IE%, which is also the measure of surface coverage with inhibitor molecules θ (IE_%_ = θ × 100%). According to the principles of the most commonly used Langmuir adsorption isotherm, the adsorption equilibrium constant, K_ads_, depends on surface coverage θ, which is given with Equation (8):

(8) $$K_{ads}c_{inh}=\left(\frac{\theta }{1-\theta }\right)$$

In the linear range of Equation (8), the adsorption isotherm may serve for calculation of the adsorption Gibbs free energy ΔG, using Equation (9):

(9) $$\Delta G=-RTln\left(K_{ads}\times 55.5\right)$$

Importantly, Langmuir isotherm conditions are only fulfilled for concentrations below full coverage with the adsorbent monolayer. One should note, that classical approaches towards adsorption isotherm determination are based on merely few measurement points, where the non-linear behavior resulting from aforementioned situation is difficult to track. On the other hand, the quasi-capacitance parameter obtained during g-DEIS impedance measurements allows estimating the exact concentration required for monolayer formation by inhibitor molecules [20]. 

Figure 6c presents the adsorption isotherms obtained with g-DEIS approach and plotted according to the Langmuir model of molecules adsorption. The isotherms were drawn for resorcinol and catechol at concentrations in range 0–10 mM. It can be pointed out that both of these functions are characterized by loss of linear character and both have inflection at concentrations between 2 and 4 mM. However, the effective capacitance C_eff_ measurements plotted in the inset of Figure 6c reveals significant differences in the adsorption mechanism. 

There are three primary factors affecting the value of capacitance according to Equation (10), namely, electrochemically active surface area A, permittivity ε, and layer thickness d:

(10) $$C=\frac{\varepsilon_{0}\varepsilon A}{d}$$

where ε_0_ is vacuum permittivity. Normalization of heterogeneity factor affecting quasi-capacitance Q and estimation of the effective capacitance C_eff_ allows ignoring the effect of electrode heterogeneity. Previous studies have shown that with relatively short measurement duration the key factor influencing instantaneous C_eff_ of the adsorbed layer is its thickness [20,38]. 

In the case of a catechol-exposed AA5754 electrode, the C_eff_ value increases until reaching its maximum at c_inh_ = 3.5 mM and then decreases. Therefore, it should be assumed that the full coverage of aluminum surface with corrosion inhibitor molecules occurs at this concentration and the following C_eff_ decrease results from the increase in adsorbed layer thickness. The adsorption isotherm still remains linear afterward, but no longer following the aforementioned restriction regarding surface coverage. 

The situation is essentially different in the case of resorcinol-exposed AA5754 electrodes, where according to classic EIS and XPS studies the inhibitory action is generally lower. In the whole studied concentration range, up to 10 mM the C_eff_ value effectively increases. The competitive interaction of the corrosion products layer and the resorcinol adsorption layer may have its effect on difficulties in assessing the full coverage of the adsorption layer but also influences layer permittivity. As a result, the adsorption isotherm for resorcinol was following Langmuir model of adsorption at significantly higher inhibitor concentrations than catechol. On the other hand, the estimated inhibitor efficiency at the lowest concentrations is negligible and thus hard to measure. A conclusion should be made that the applicability of the Langmuir adsorption isotherm model for catechol and resorcinol lies in different inhibitor concentration ranges. 

Following Equations (8) and (9), the calculated values of Gibbs free energy ΔG of the adsorption process are presented in Table 3. Their negative values in both cases confirm spontaneous adsorption of both studied dihydroxybenzene molecules on aluminum alloy 5754 surface. Nevertheless, the significantly different ΔG values between resorcinol and catechol suggest an altered adsorption mechanism. 

The more negative Gibbs free energy values are typical in case of chemisorption and formation of chemical bonds between filled π-orbitals in the oxygen atoms and partially unoccupied π-orbitals in the d-block metals. This is the postulated adsorption mechanism of resorcinol. Naturally, the value of this thermodynamic parameter may be further influenced by reported presence of the nonstoichiometric corrosion products layer. On the other hand, our previous studied on carboxylic acids revealed that the less negative free Gibbs energies correspond not only to the electrostatic interaction of the physisorption process but also the ligand exchange model of adsorption, resulting in formation of coordination compounds at the metal interface [19,20]. This is the case of catechol interaction. The lower efficiency of ligand formation by resorcinol and quinol originates from the molecule geometry.

### 3.4. The Keto-Enol Tautomerism

**Figure 7:**
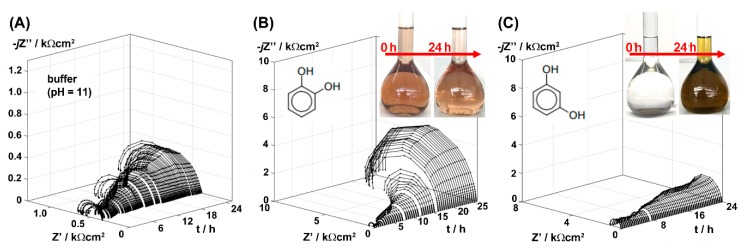
The g-DEIS impedance graphs of aluminum alloy 5754 in Nyquist projection during 24 h exposure in (**A**) bicarbonate buffer (pH = 11); and with the addition of (**B**) 10 mM catechol and (**C**) 10 mM resorcinol.

During the long-term exposure tests, the authors observed changes in the color of the studied electrolytes over time, and resorcinol in particular (see inset of Figure 7). These changes were followed by alteration of the electrochemical characteristics over time. On the other hand, for catechol and quinol the long-term inhibition efficiency was significantly higher. The g-DEIS studies were carried out once more to track the exact change in the electrochemical behavior of aluminum alloy 5754 during 24 h exposure. The results of the impedance monitoring are presented in Figure 7.

Analysis of the impedance data makes it possible to draw conclusions regarding the long-term behavior of AA5754 under the studied electrolytic conditions. The slight increase of the semicircle diameter over time, seen on the Nyquist projection for buffer-exposed sample, makes it possible to conclude that the corrosion product layer forming on metal surface provides partial barrier properties and decreases the corrosion rate approximately 2.5×. 

When exposed to buffer with addition of 10 mM catechol, the instantaneous charge transfer resistance is slightly higher (typically around 0.4 kΩ) and then gradually increases over time to reach significantly improved inhibition efficiency of ~98% after 24 h exposure. On the other hand, AA5754 exposed to electrolyte containing the same amount of resorcinol shows very small increase of charge transfer resistance over duration of the long-term exposure experiment.

The long-term exposure study allows drawing two important conclusions. First, there must be an additional interaction between studied inhibitor molecules and the electrolyte or the analyzed sample, which further differentiates the electrochemical characteristics of these dihydroxybenzene isomers over time. Second, when performing inhibitor efficiency measurements one has to take into consideration possible changes of investigated system characteristics. This is possible by carrying out fast measurements with techniques that allow non-stationary process analyses (such as g-DEIS). Alternatively, one could employ a sufficiently long conditioning period, which might be different for each studied system. The latter approach, although more accessible, may cause problems in terms of meeting the conditions for many adsorption models.

The authors claim that the mechanism leading to further differentiation of adsorption by catechol and resorcinol on the aluminum alloy surface is the keto-enol tautomerism, which may occur in aqueous alkaline environments. Nuclear magnetic resonance (NMR) studies were performed in order to verify this hypothesis. 

**Figure 8:**
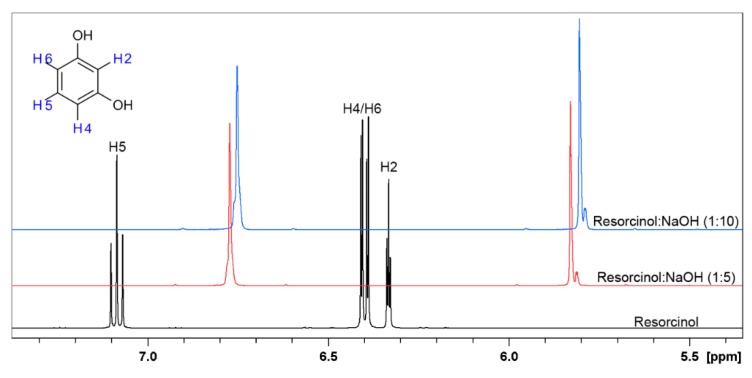
^1^H-NMR spectra of resorcinol in D_2_O (black line) and in the presence of NaOH in D_2_O in the molar ratio 1:5 (red line) and 1:10 (blue line), respectively.

^1^H-NMR measurements were performed in order to determine the presence of possible keto-enol forms in resorcinol in the alkaline conditions or to determine the formation of a salt of those compounds. The studies of proton transfer by ^1^H-NMR titration present a useful technique to determine the keto-enol equilibria [62,63]. The ^1^H-NMR spectra of resorcinol dissolved in D_2_O with addition of NaOH in molar ration of 1:5 and 1:10 are presented on Figure 8. The ^1^H-NMR spectra of resorcinol have previously been performed in D_2_O [64,65], while the titration of this compound by NaOH has not been investigated. 

Two triplets are observed on spectra of resorcinol in D_2_O, which correspond to H5 and H2, while two doublets correspond to H4 and H6. The spectra of resorcinol after the addition of NaOH in molar ratio 1:5 changed diametrically. The shape, the chemical shifts and the multiplicity are different in comparison to the first one. Two main signals shifted towards negative values are now observed. This phenomenon indicates that the protons present in the structure of resorcinol are changed, further influencing the chemical shifts and the multiplicity. The addition of molar excess of NaOH in (1:10), does not cause any additional changes in ^1^H-NMR spectrum shape, but the peaks are further shifted. 

**Figure 9:**
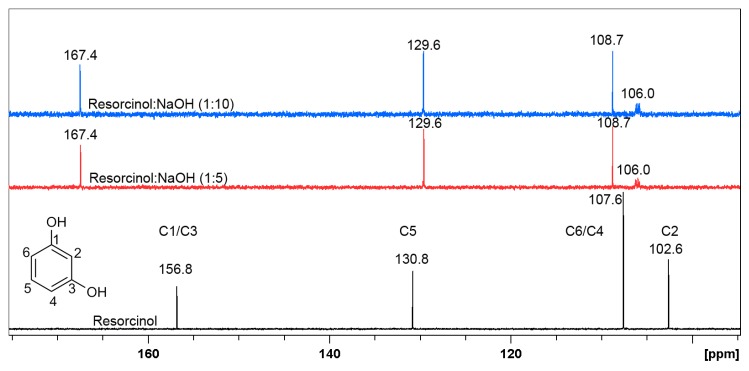
^13^C-NMR spectra of resorcinol in D_2_O (black line) and in the presence of NaOH in D_2_O in molar ratio 1:5 (red line) and 1:10 (blue line), respectively.

The determination of the new resorcinol derivative structure was possible after measuring the ^13^C-NMR spectra, shown on Figure 9. These spectra were performed in D_2_O and after addition of NaOH in molar ratio 1:5 and 1:10, similar to previous experiment. It should be noticed that the peaks C4, C5, and C6 present in spectra before and after addition of NaOH do not change their position significantly. On the other hand, peaks C1 and C3, overlapping at 156.8 ppm, change their position to 167.4 ppm with the addition of NaOH, which may indicate that keto form is present in the structure of resorcinol regardless the molar ratio. Additionally, after addition of NaOH the shift of peak C2 is observed from 102.6 ppm to 106.0 ppm, this shift confirms the formation of keto form.

**Figure 10:**
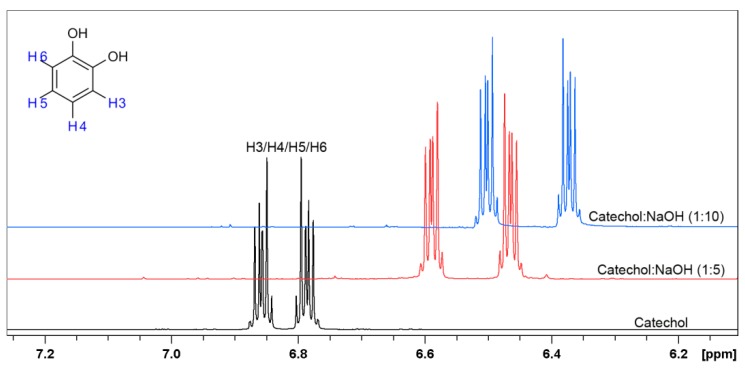
^1^H-NMR spectra of catechol in D_2_O (black line) and in the presence of NaOH in D_2_O in molar ratio 1:5 (red line) and 1:1 (blue line), respectively.

In the next step, the ^1^H-NMR and ^13^C-NMR spectra were performed for catechol under the same experimental conditions. The ^1^H-NMR spectra of catechol have previously been performed in aqueous solution, but under acidic pH = 2.4 [66] and in CDCl_3_ [67]. The shape and chemical shifts of ^1^H-NMR spectra illustrated on Figure 10 are very similar to those in the literature.

Two multiplets are present regardless of the solution, while the addition of NaOH in ratio 1:5 and 1:10, respectively, do not cause any changes in ^1^H-NMR spectra shape; however, with increasing molar ratio of NaOH, the spectra are shifting towards more negative values. The above changes clearly indicate that the chemical structure of the catechol does not change significantly, while the shifts may indicate formation of sodium salts of catechol [68].

**Figure 11:**
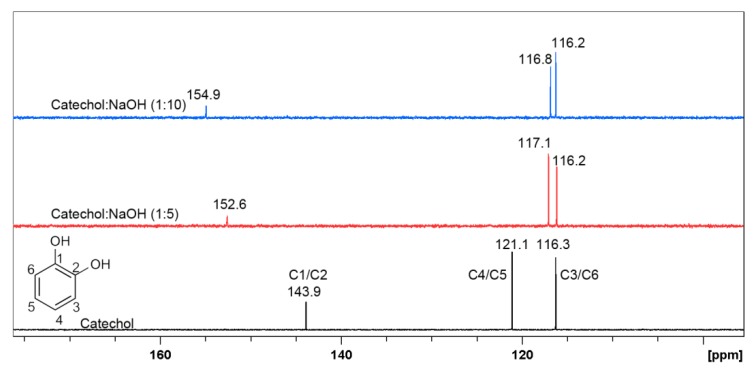
^13^C-NMR spectra of catechol in D_2_O (black line) and in the presence of NaOH in D_2_O in the molar ratio 1:5 (red line) and 1:1 (blue line), respectively.

^13^C-NMR spectra were performed in order to confirm the formation of sodium salt of catechol in alkaline conditions, as shown on Figure 11. The obtained data confirm previously drawn assumptions. ^13^C-NMR spectra reveal ^13^C chemical shifts for catechol in D_2_O with the presence of NaOH in molar ratio 1:5 and 1:10. The C1 and C2 atoms connected to the hydroxyl groups in catechol in investigated solutions both give signal at 143.9 ppm in absence of NaOH, but after its addition, the signal is shifted towards 152.6 ppm and 154.9 ppm for ratio 1:5 and 1:10, respectively. The shift of carbon signals directly indicates that catechol in alkaline solutions forms a salt [69]. It is worth noticing that a small change of peak position was also observed for carbon C4/C5 and C3/C6 after additions of NaOH.

### 3.5. Dihydroxybenzene Isomers Adsorption Mechanism

**Figure 12:**
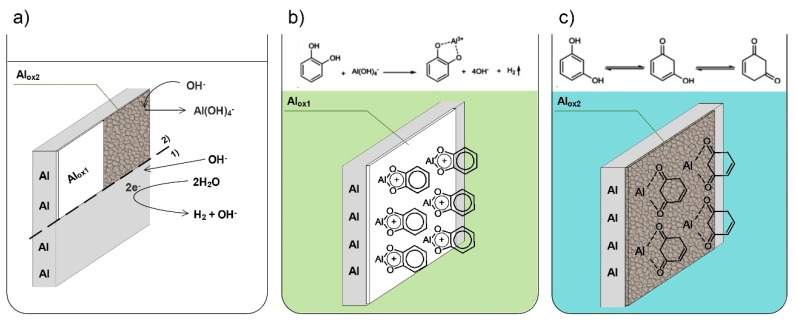
Schematic representation of aluminum corrosion mechanism in aqueous alkaline solutions: (**a**) in absence of corrosion inhibitor and with the addition of (**b**) catechol; (**c**) resorcinol. Phase (1) represents the OH^−^ attack leading to formation Al(OH)_3_, phase (2) describes the interaction between OH^−^ and Al(OH)_3_, leading to the formation of Al(OH)_4_^−^ and a non-stoichiometric insoluble corrosion product layer.

The overall interaction of the studied dihydroxybenzene isomers with aluminum alloy 5754 surface may thus be explained using the scheme presented on Figure 12. Figure 12a illustrates the case of aluminum corrosion in an alkaline environment, according to the two-step mechanism discussed in the introduction section: 1) attack of OH^−^ ions on Al_2_O_3_ leading to its dissolution and Al(OH)_3_ formation, followed shortly after by 2) chemical formation of Al(OH)_4_^−^ ions. The corrosion inhibition mechanism for catechol considers formation of ligands with aluminum ions (see Figure 12b), which is hindered in the case of resorcinol. On the other hand, resorcinol depends on the formation of insoluble corrosion products layer, which to a large extent provides a barrier mechanism towards corrosive electrolyte. The molecules chemisorb on the corrosion products layer, which becomes very efficient only at high inhibitor concentrations. 

The lower inhibition efficiency of resorcinol at concentrations not exceeding 10 mM is connected with the keto-enol tautomerism mechanism, occurring in aqueous alkaline media and lowering the molecule influence on the corrosion protection (see Figure 12c). Due to local differences in pH in anodic and cathodic zones of electrode/electrolyte interface, the dynamics of the keto-enol tautomerism may be locally altered. The NMR spectra revealed that the process takes place in wide pH range.

## 4. Conclusions

Understanding the interaction mechanism of inhibitor molecules with the protected metal surface is of key importance in the selection of the most efficient corrosion inhibitors, the most important, in particular, in the case of green inhibitors based on natural extracts. While it is widely known how different functional groups affect the adsorption mechanism, the differences introduced by its location within the inhibitor molecule are often omitted. 

In this study, we revealed how the position of hydroxyl groups affects the adsorption mechanism of dihydroxybenzene isomers and offered corrosion resistance toward alumnium alloy 5754 surface in alkaline environment. The utilization of Dynamic Electrochemical Impedance Spectroscopy in galvanostatic mode (g-DEIS) for adsorption isotherm determination made it possible to confirm different forms of dihydroxybenzene interaction. All of the studied inhibitors followed the Langmuir model of adsorption, although we have observed that its applicability lies in different inhibitor concentration ranges.

Resorcinol was found to be characterized by the chemical adsorption mechanism. Its adsorption on aluminum surface is competitive to insoluble corrosion product layer formation, as shown with SEM and XPS studies. This interaction leads to the best inhibitor efficiency at the highest investigated concentration of 100 mM, but is not as efficient at lower concentrations. On the other hand, catechol and quinol follow the ligand exchange model of adsorption. This leads to more efficient adsorption and increases corrosion protection even at lower corrosion concentrations: 1 and 10 mM. The adsorption process dominates insoluble corrosion product layer formation, the presence of which on the analyzed surface was negligible.

The next significant difference lies in the long-term behavior and corrosion protection offered by dihydroxybenzene isomers in alkaline electrolyte. We report that resorcinol molecules undergo keto-enol tautomerism in sodium hydroxide solution, while the aforementioned process was negligible in the case of quinol and catechol. The tautomerism leads to the rebuilding of the inhibitor molecule, electrolyte discoloration, but does not have significant influence on the chemical adsorption mechanism by resorcinol over longer periods of time. It is even possible that the presence of keto-enol tautomerism itself is the reason behind hindered adsorption of resorcinol and offered corrosion resistance. Keto forms were not observed in the structure of catechol and quinol molecules. At the same time, their ability to complex metal ions leads to formation of layers with higher barrier properties and increased corrosion inhibition.

## Figures and Tables

**Table 1:** Electric parameters of the studied systems obtained from EIS results fitted with R(QR) EEC.

C_inh_/mM	Buffer	Resorcinol			Catechol			Quinol		
	-	1	10	100	1	10	100	1	10	100
Q/µSs^α^·cm^−2^	23.9	22	29.4	14.5	24.2	31.1	19	25.5	28	14.5
α/-	0.94	0.94	0.92	0.94	0.91	0.89	0.9	0.93	0.93	0.96
C_eff_/µF·cm^−2^	10.7	10	10.8	8.2	7.5	7.6	6.5	10.7	11.8	9.4
R_ct_/kΩ·cm^2^	0.14	0.18	0.3	10.93	0.29	0.38	3.64	0.39	0.35	2.45
IE_%_/%	-	22.2	53.3	98.7	51.7	63.2	96.2	64.1	60	94.3

**Table 2:** Surface chemical composition (in at.%) for AA5754 after 24 h exposure in bicarbonate buffer (pH = 11) with the addition of resorcinol or catechol, based on high-resolution XPS analysis.

Chemical State		BE/eV	Bicarbonate Buffer	Resorcinol		Catechol	
				10 mM	100 mM	10 mM	100 mM
Al2p_3/2_	Al_ox1_	74.8	1.5	3.2	2.3	3.4	8.0
	Al_ox2_	76.7	19.8	14.7	15.3	10.5	-
C1s	C-C	284.7	1.4	4.6	5.4	10.4	6.0
	C-OH	286.0	12.1	12.8	16.9	31.4	32.8
	C=O	287.6	2.3	7.6	5.1	3.9	6.1
	COOH	290.3	1.6	2.8	2.4	3.9	8.6
O1s	O^2−^	531.3	30.8	20.0	22.1	12.6	3.4
	CO/OH	532.6	16.4	15.2	18.5	20.2	29.2
	C=O/H_2_O	533.7	13.0	18.0	11.0	3.0	5.6
Mg1s	Mg_ox_	1303.1	1.1	1.1	1.0	0.7	0.3

**Table 3:** The applicability range of the Langmuir adsorption model and the obtained thermodynamic parameters for resorcinol and catechol.

Isomer	Langmuir Model c_inh_ Range/mM	Adsorption Equilibrium Constant K_ads_	Gibbs Free Energy ΔG/kJmol^−1^
catechol	<3.5	1.39	−10.77
resorcinol	2–10	11.38	−15.98
